# Neuroglia infection by rabies virus after anterograde virus spread in peripheral neurons

**DOI:** 10.1186/s40478-020-01074-6

**Published:** 2020-11-23

**Authors:** Madlin Potratz, Luca M. Zaeck, Carlotta Weigel, Antonia Klein, Conrad M. Freuling, Thomas Müller, Stefan Finke

**Affiliations:** Federal Research Institute for Animal Health, Institute of Molecular Virology and Cell Biology, Friedrich-Loeffler-Institut (FLI), 17493 Greifswald-Insel Riems, Germany

**Keywords:** Rabies pathology, Tissue optical clearing, 3D tissue imaging, Light sheet microscopy, Neuroglia infection, Schwann cell

## Abstract

The highly neurotropic rabies virus (RABV) enters peripheral neurons at axon termini and requires long distance axonal transport and trans-synaptic spread between neurons for the infection of the central nervous system (CNS). Recent 3D imaging of field RABV-infected brains revealed a remarkably high proportion of infected astroglia, indicating that highly virulent field viruses are able to suppress astrocyte-mediated innate immune responses and virus elimination pathways. While fundamental for CNS invasion, in vivo field RABV spread and tropism in peripheral tissues is understudied. Here, we used three-dimensional light sheet and confocal laser scanning microscopy to investigate the in vivo distribution patterns of a field RABV clone in cleared high-volume tissue samples after infection via a natural (intramuscular; hind leg) and an artificial (intracranial) inoculation route. Immunostaining of virus and host markers provided a comprehensive overview of RABV infection in the CNS and peripheral nerves after centripetal and centrifugal virus spread. Importantly, we identified non-neuronal, axon-ensheathing neuroglia (Schwann cells, SCs) in peripheral nerves of the hind leg and facial regions as a target cell population of field RABV. This suggests that virus release from axons and infected SCs is part of the RABV in vivo cycle and may affect RABV-related demyelination of peripheral neurons and local innate immune responses. Detection of RABV in axon-surrounding myelinating SCs after i.c. infection further provided evidence for anterograde spread of RABV, highlighting that RABV axonal transport and spread of infectious virus in peripheral nerves is not exclusively retrograde. Our data support a new model in which, comparable to CNS neuroglia, SC infection in peripheral nerves suppresses glia-mediated innate immunity and delays antiviral host responses required for successful transport from the peripheral infection sites to the brain.

**Electronic supplementary material:**

The online version of this article (10.1186/s40478-020-01074-6) contains supplementary material, which is available to authorized users.

## Introduction

Rabies is estimated to cause 59,000 human deaths annually in over 150 countries, with 95% of cases occurring in Africa and Asia (reviewed in [21, 26]). The disease is caused by a plethora of highly neurotropic, non-segmented, single-stranded RNA viruses of negative polarity belonging to the family *Rhabdoviridae* of the order *Mononegavirales,* of which the rabies virus (RABV) is the type species of the *Lyssavirus* genus [2]. RABV and other lyssaviruses enter the nervous system at the synapses of peripheral neurons. After receptor-mediated endocytosis, the virions remain in endosomal vesicles and hijack the retrograde axonal transport machinery [23, 34, 60] to reach the neuron soma, where virus ribonucleoproteins (RNPs) are released into the cytoplasm [48] for virus gene expression and replication. Newly formed virus is then transported to post-synaptic membranes, followed by trans-synaptic spread to connected neurons [12]. Prolonged replication of pathogenic RABV in the brain eventually leads to the onset of a progressive rabies encephalitis, invariably resulting in fatal disease progression. In animals suffering from RABV encephalitis, the virus can be detected in multiple nervous or innervated tissues including hind leg, spinal cord, brain, face, and salivary glands [5, 15]. For the transmission of infectious virus to new hosts via virus-containing saliva, centrifugal spread of RABV in the peripheral nervous system (PNS) is required [18]. The secretion of RABV-containing saliva by salivary glands is commonly accepted as the main source of virus shedding and virus transmission to animals via bites (reviewed in [16, 21]).

Detection of RABV in sensory neurons of the olfactory epithelium and other tissues during late stages of infection indicates that, at least in these late phases of disease, the axonal transport is not exclusively retrograde (reviewed in [16]). In fact, studies have shown that RABV enters the anterograde axonal transport pathway in cultivated primary rat dorsal root ganglion (DRG) neurons [6] and in mice [71]. However, the consequences of anterograde transport of RABV is still controversially discussed, even though infection through intradermal sensory neurons represents a likely route of entry for lyssaviruses as a result from bat bites [7].

Because of their trans-synaptic spread, RABVs constitute ideal viruses to map neuronal connections [67, 68] as even attenuated RABVs enter axonal transport pathways [34]. More neuroinvasive yet still lab-adapted strains like CVS-11 have been used as polysynaptic neuron tracers and the resultant insights have subsequently been used to explain in vivo RABV spread and pathogenesis [62]. However, already between different CVS strains, substantial differences in pathogenicity exist [40], raising the question whether knowledge from established lab-adapted strains can be extrapolated to explain actual field RABV infection and pathogenesis.

The advent of efficient full-genome cloning techniques opened novel avenues for the analysis of virus variability in combination with phenotypical characterization of recombinant field viruses at a clonal level [44]. For example, a recent comparison of field and lab RABV clones in mice revealed that field viruses not only showed a higher virulence compared to lab strains CVS-11, ERA and SAD L16 but also substantially differ from these viruses in their ability to establish infection in non-neuronal astroglia in the central nervous system (CNS) [49]. In contrast to attenuated lab-adapted RABV, which is supposed to be eliminated from infected glial cells (astrocytes) by type I interferon-induced immune reactions [47], it has been hypothesized that rapid onset of field virus gene expression in non-neuronal astrocytes blocks inhibitory innate immune activation and responses [49]. This may not only affect RABV replication in glial cells directly but also in surrounding neurons via paracrine signalling. Thus, this mechanism may be crucial to RABV spread and pathogenesis in vivo. Apart from astrocytes, in vivo infection of non-neuronal muscle, epidermal, and epithelial cells have been described [5, 12, 13, 43]. In foxes, which had been orally immunized with live-attenuated RABV, infected cells can be detected in the epithelium of lymphoid tonsils [57, 64]. In vitro, even immature dendritic cells (DCs) or monocytes can be infected [36]. Overall, knowledge on the broad spectrum infection pattern of RABV in vivo, particularly of field RABV strains, is still incomplete and the identification of PNS and non-neuronal cells involved in RABV spread and pathogenesis has been given little attention.

Therefore, by using a highly virulent field RABV clone of dog origin (rRABV Dog) [44], we set out to systematically and comprehensively elucidate the in vivo spread and cell tropism of a field RABV strain. So far, technical limitations in conventional histology to visualize infection processes in complex tissues in large three-dimensional (3D) volumes at a mesoscopic scale and high resolution hindered a comprehensive overview of RABV cell tropism, in vivo spread, and pathogenesis. Immunofluorescence-compatible tissue clearing iDISCO and uDISCO (immunolabeling-enabled and ultimate three-dimensional imaging of solvent-cleared organs, respectively) protocols [46, 50] recently allowed us to perform high-resolutions 3D immunofluorescence analyses of thick RABV-infected tissues slices [49, 70].

Here, we investigated the in vivo spread of a highly virulent field RABV clone (rRABV Dog) through the CNS and PNS after intramuscular (i.m.) and intracranial (i.c.) inoculation. Using 3D immunofluorescence imaging of solvent-cleared tissues by light sheet and confocal laser scanning microscopy, we identified RABV infections in hind leg nerves, spinal cord, brain, and nerves of the facial region. These data provide a broad overview of field RABV distribution in the clinical phase of rabies encephalitis. Notably, we were able to identify Schwann cells (SCs) as a so far unrecognized non-neuronal RABV target cell population, which may be involved in regulating RABV infection and, thus, pathogenesis in peripheral nerve tissues. Moreover, infection of SCs after centrifugal RABV spread provides evidence for anterograde axonal transport and infectious virus release at distal axon membrane sites of peripheral neurons.

## Materials and methods

### Viruses

The recombinant field virus clone rRABV Dog [44] was derived from an isolate originating from an infected dog from Azerbaijan, which was used in a challenge study [65]. RABV Bat (FLI virus archive ID N 13240) was isolated from an insectivorous North American bat.

### Animal trials

Six- to eight-week-old BALB/c mice (Charles-River, Germany) were i.m.-infected with decreasing doses of rRABV Dog. Four groups of six animals each were anesthetized with isoflurane and infected with 3 × 10^0^, 3 × 10^1^, 3 × 10^2^ and 3x10^3^ TCID_50_/mouse by injection of 30 µL of virus suspension in the femoral hind leg muscle. Additionally, three mice were i.c.-infected at a dose of 1 × 10^2^ TCID_50_/mouse. Body weight and clinical scores from one to five (1: ruffled fur, hunched back; 2: slowed movement, circular motions, weight loss > 15%; 3: tremors, shaky movement, seizures, weight loss > 20%; 4: paralysis, weight loss > 25%; 5: coma/death) were determined daily until day 21 post-infection or day of euthanasia. After reaching a clinical score of two, the animals were anaesthetized with isoflurane and euthanized by decapitation. Samples were taken, fixed with 4% paraformaldehyde (PFA) for 1 week, and stored for further processing. All remaining animals were euthanized at 21 dpi. The animal approaches were evaluated by the responsible animal care, use, and ethics committee of the State Office for Agriculture, Food Safety, and Fishery in Mecklenburg-Western Pomerania (LALFF M-V) and gained approval with permission 7221.3-2-047/19. For RABV Bat, archived tissue samples (hind legs, spinal columns, and heads) from previous virus characterization experiments were used (LALFF M-V permission 7221.3-2.1-001/18).

### Detection of RABV RNA in oral swabs and salivary glands via RT-PCR

Salivary gland samples were homogenized in 1 ml cell culture medium in the UPHO Ultimate Homogenizer (Geneye, Hong Kong) using a 3 mm steal bead. Oral swab samples were transferred into 1 ml of culture medium. Total RNA from oropharyngeal swabs as well as from salivary gland tissues were extracted using the NucleoMagVet kit (Macherey & Nagel, Germany) according to manufacturer’s instructions in a KingFisher/BioSprint 96 magnetic particle processor (Qiagen, Germany). RABV RNA was detected by the R14-assay, a RT-qPCR targeting the N-gene [27]. The RT-qPCR reaction was prepared using the AgPath-ID-One-Step RT-PCR kit (Thermo Fisher Scientific, USA) adjusted to a volume of 12.5 µl [19]. RT-qPCRs were performed on a BioRad real-time CFX96 detection system (Bio-Rad, USA).

### Antibodies and dyes

To detect RABV in the tissues, a polyclonal rabbit serum against recombinant RABV P protein (P160-5; 1:3000 in PTwH [0.2% Tween 20 in PBS with 10 µg/mL heparin]) was used [45]. The following first antibodies and dyes were obtained from the respective suppliers: chicken anti-NEFM (Thermo Fisher, USA; #PA1-16758, RRID:AB_2282551; 1:1000 in PTwH), guinea pig anti-NEFM (Synaptic Systems, Germany; #171204, RRID:AB_2619872; 1:400 in PTwH), chicken anti-MBP (Thermo Fisher; #PA1-10008, RRID:AB_1077024; 1:500 in PTwH), TO-PRO™-3 iodide (Thermo Fisher; #T3605; 1:1000 in PTwH.) As secondary antibodies donkey anti-rabbit Alexa Fluor^®^ 568 (Thermo Fisher; #A10042, RRID:AB_2534017), donkey anti-chicken Alexa Fluor^®^ 488 (Dianova, Germany; #703-545-155, RRID:AB_2340375), donkey anti-guinea pig Alexa Fluor^®^ 647 (Dianova; #706-605-148, RRID:AB_10895029) were used, each at dilution of 1:500 in PTwH.

### iDISCO-based immunostaining and uDISCO-based clearing of tissue samples

Staining and clearing protocols were adapted from earlier reports [46, 50] and were performed as described previously [49, 70]. Hind legs were skinned prior to fixation. Prior to the slicing, PFA-fixed hard tissues, which included hind legs, spinal columns, and heads, were decalcified with 20% ethylenediaminetetraacetic acid (EDTA) [w/v] in H_2_O_dd_ (pH was adjusted to 7.0) for 4 days at 37 °C. The EDTA solution was exchanged daily. Afterwards the decalcified tissues were cut into 1 mm thick sections using a scalpel. For brains, as soft tissues, a vibratome (VT1200S, Leica Biosystems, Germany) was used. After treatment with increasing methanol concentrations in distilled water (20%, 40%, 60%, 80%, and twice 100%) and bleaching with 5% H_2_O_2_ in pure methanol at 4 °C, the samples were rehydrated with decreasing concentrations of methanol in distilled water (80%, 60%, 40%, and 20%). After washing with PBS and 0.2% Triton X-100 in PBS, the samples were permeabilized for 2 days at 37 °C in 0.2% Triton X-100/20% DMSO/0.3 M glycine in PBS. After subsequent blocking with 6% donkey serum/0.2% Triton X-100/10% DMSO in PBS at 37 °C for another 48 h, primary antibodies in PTwH were added for 5 days with refreshment of the antibody solution after 2.5 days. The samples were washed with PTwH four times until the next day, when secondary antibodies in PTwH were added for 5 days with refreshment of the antibody solution after 2.5 days. After further washing for four times until the next day, the tissues were dehydrated in a series of *tert*-butanol (TBA) solutions (from 30% to 96% in distilled water), each step for 2 h (except for over night incubation with 80% TBA), final incubation in 100% TBA and subsequent clearing in BABB-D15 with 0.4 vol% DL-α-tocopherol until they were optically transparent (2–6 h). BABB-D15 is a combination of benzyl alcohol (BA) and benzyl benzoate (BB) at a ratio of 1:2, which is mixed at a ratio of 15:1 with diphenyl ether (DPE). The samples were either analyzed directly by light sheet microscopy or were transferred to 3D-printed imaging chambers as described before [70].

### Light sheet and confocal laser scanning microscopy

For light sheet microscopy, a LaVision UltraMicroscope II with an Andor Zyla 5.5 sCMOS Camera, an Olympus MVX-10 Zoom Body (magnification range: 0.63–6.3x), and an Olympus MVPLAPO 2 × objective (NA = 0.5) equipped with a dipping cap was used. Z-stacks were acquired with a light sheet NA of 0.156, a light sheet width of 100%, and a z-step size of 2 µm. LaVision BioTec ImSpector Software (v7.0.127.0) was used for image acquisition.

Confocal laser scanning microscopy was performed with a Leica DMI 6000 TCS SP5 equipped with a HC PL APO CS2 40× water immersion objective (NA = 1.1; Leica, Germany; #15506360), using the Leica Application Suite Advanced Fluorescence software (V. 2.7.3.9723) and a z-step size of 0.5 µm.

### Image processing

For image processing, a Dell Precision 7920 workstation was used (CPU: Intel Xeon Gold 5118, GPU: Nvidia Quadro P5000, RAM: 128 GB 2666 MHz DDR4, SSD: 2 TB). Acquired image stacks were processed using the ImageJ (v1.52 h) distribution package Fiji [54, 55] or arivis Vision4D (arivis, Germany; v3.2.0). Brightness and contrast were adjusted for each channel and, if necessary, channels were denoised. Either maximum-z-projections or volumetric 3D-projections are shown.

## Results

### Field virus clone rRABV Dog undergoes the complete RABV infection cycle in adult BALB/c mice

Mouse models differ in their age-dependent susceptibility to fixed RABV lab strains and field isolates [11]. To confirm the field virus character of rRABV Dog and its suitability for investigation of the complete RABV in vivo cycle from infected muscle at the site of entry to secretion in saliva in adult mice, six- to eight-week-old BALB/c mice were inoculated by the i.m. route (hind leg muscle) with decreasing infectious virus doses. Although mortality was dose-dependent, even very low doses (30 TCID_50_/mouse) of rRABV Dog were sufficient to cause rabies disease and release of infectious virus in nearly all inoculated mice, thus, highlighting the high virulence of this field virus RABV clone. Comparable to field RABV isolates, even at an extremely low infection dose (3 TCID_50_/mouse), the complete in vivo cycle had been undergone by animals that succumbed to infection, as shown by detection of viral RNA in both salivary glands and in oral swabs using RT-qPCR (Table 1; Additional file 1: Fig. S1).

**Table 1:** Dose-dependent onset of clinical rabies signs after i.m. application of rRABV Dog in the upper hind leg muscle of 6- to 8-week old BALB/c mice

Virus dose	Euthanized	Salivary gland	Oral swab
TCID50/mouse	(Humane endpoint reached)	Virus RNA	Virus RNA
3000	6/6	6/6	5*/6
300	5/6	5/6	5/6
30	5/6	5/6	5/6
3	2/6	2/6	2/6

Salivary gland and oral swabs were virus RNA positive even at low infection doses. *One oral swab not tested

### RABV infects peripheral neuron-associated neuroglia in the hind leg after i.m. inoculation

**Fig. 1:**
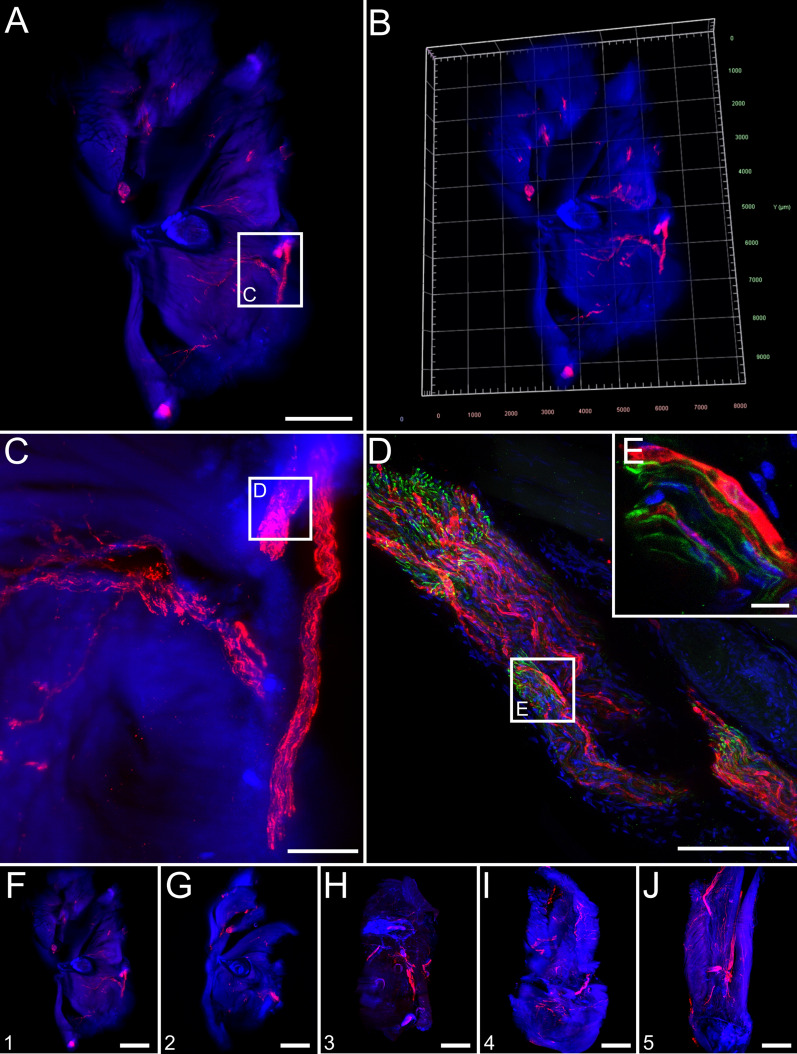
Overview of field RABV infection in mouse hind leg cross sections after i.m. infection (9 days post-inoculation). **a** Maximum z-projection of light sheet overview of a hind leg cross section [1.6× magnification; z = 1156 µm]. Red: RABV P; blue: nuclei. Green fluorescence for NEFM not shown as separation from green autofluorescence was not possible at low resolution. Scale bar: 1500 µm. **b** 3D projection of (**a**). **c** Maximum z-projection of detail (white box in **a**) with infected nerve [12.6× magnification; z = 574 µm]. Scale bar: 200 µm. **d** Maximum z-projection of confocal z-stack of indicated region in (C) [z = 48 µm] of infected nerve with NEFM signal (green). Scale bar: 100 µm. Note: due to the separate image acquisition, the orientation of the shown nerve is different to (**c**). **e** Maximum z-projection of detail from (**d**) [z = 10 µm]. Scale bar: 10 µm. **f–j** Maximum z-projections of serial thick hind leg sections from the upper leg (**f**) to the knee (**j**) [1.6× magnification, z = 1156 µm (**f**), 1402 µm (**g**), 1172 µm (**h**), 1990 µm (**i**), 1372 µm (**j**)]. For reference, see indicated slices in Additional file 1: Fig. S2A. For improved intelligibility, only RABV P (red) and nuclei (blue) are shown. Scale bar: 1500 µm

Immunostaining of solvent-cleared hind leg slices of diseased animals for RABV phosphoprotein (P), neurofilament M (NEFM), and cell nuclei (TO-PRO™-3) was performed for light sheet and confocal laser scanning microscopy analysis (Fig. 1). RABV infection of peripheral nerves innervating the femoral tissue could be demonstrated (Fig. 1a–c, red). Reconstruction of a 3D femoral tissue volume allowed visualization of the distribution of infected nerve fibres in their spatial environment (Fig. 1b, Additional file 2: Video S1). The specificity of the RABV immunofluorescence was confirmed by using non-infected hind leg tissues as negative controls (Additional file 1: Fig. S3). At a higher resolution, axons were identified via staining by the neuronal cytoskeleton marker (NEFM) (Fig. 1d, e, green) but, notably, did not co-localize with RABV P (Fig. 1e). As RABV-infected peripheral nerves could be visualized in all serial femoral slices analyzed (Fig. 1f–j), our approach proved to be appropriate to reconstruct a 3D model of RABV infection patterns in peripheral tissues.

**Fig. 2:**
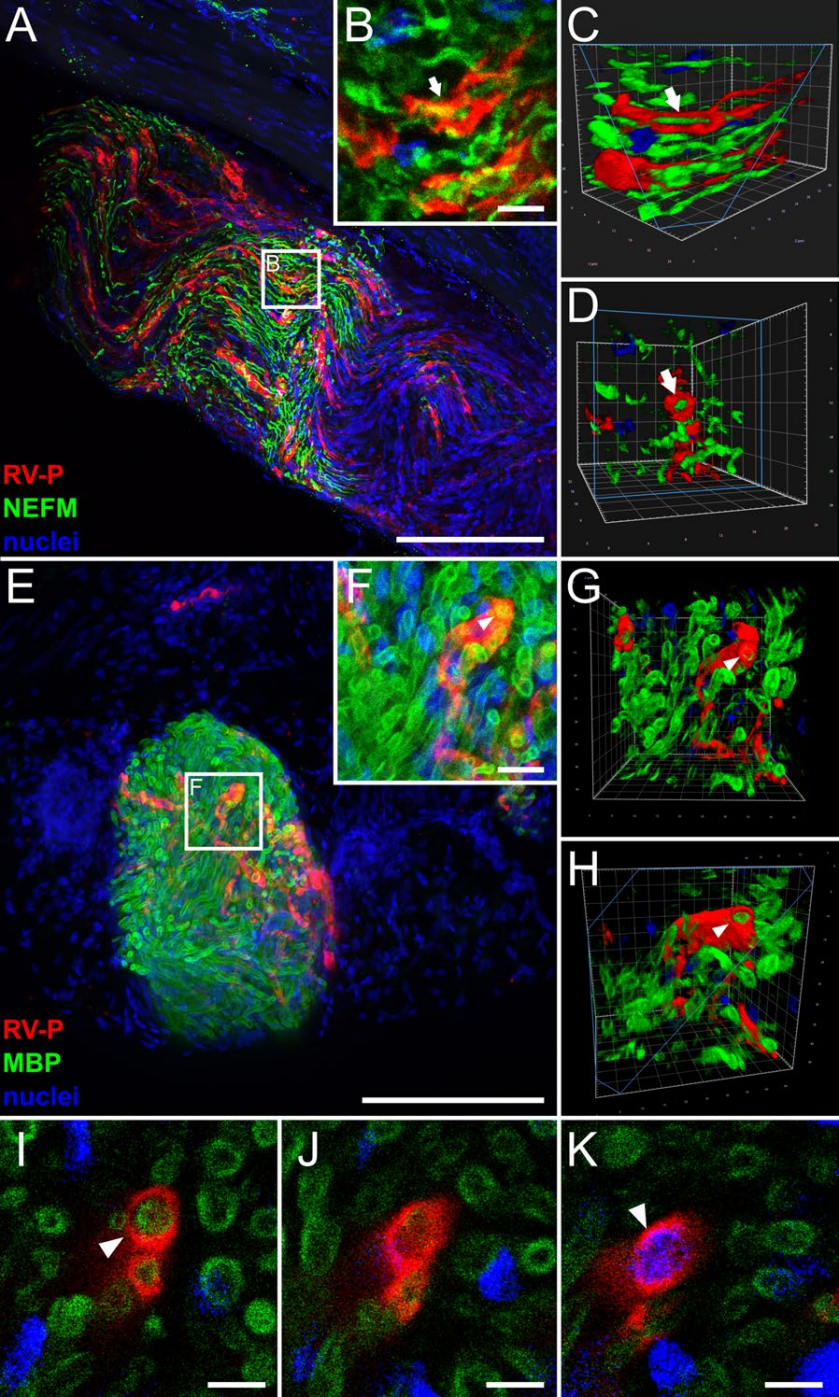
Detection of RABV P in non-neuronal Schwann cells of hind leg tissue after i.m. inoculation (9 days post-inoculation). **a**, **b** Maximum z-projection of high-resolution confocal z-stacks of RABV-infected nerve fiber (see also Fig. 1d) in hind leg sections [z = 45 µm; Scale bar: 100 µm] (**a**) and detail [z = 15 µm; Scale bar: 10 µm] (**b**). Red: RABV P; green: NEFM; blue: cell nuclei. **c**, **d** Volumetric 3D projections of (**b**) with different viewing angles. The light blue box indicates the clipping plane. Arrows: RABV P surrounding axonal NEFM. **e**, **f** Maximum z-projection of a hind leg section (**e**) [z = 134 µm; Scale bar: 200 µm] and detail (**f**) [z = 20 µm; Scale bar: 10 µm] stained for RABV P (red), MBP (green) and nuclei (blue). **g**, **h** Volumetric 3D projections of different viewing angles of (**f**). Arrowhead: RABV-infected Schwann cell **i–k** Single slices with RABV P (red), MBP (green) and nuclei (blue). Arrowheads indicate myelin sheath/nucleus surrounded by RABV P. Scale bar: 5 µm

Further confocal laser scanning analysis confirmed the lack of co-localization of RABV P with NEFM in axons (Fig. 2a, b). Rather, RABV P-specific signals were arranged next to or around the tubular NEFM signals (Fig. 2c, d, arrows; Additional file 3: Video S2). Similar spatial arrangement patterns were observed after staining for the Schwann cell (SC) marker myelin basic protein (MBP) (Fig. 2e–k). MBP is known to be part of the myelin sheath of SCs that forms tubular structures around the axons (reviewed in [52]) and accordingly, appeared as hollow tubules that were surrounded by RABV P (Fig. 2g–k, arrowheads; Additional file 4: Video S3). Corroborated by the presence of nuclei in RABV-infected cells (Fig. 2k), this suggests that RABV P accumulated in the cytoplasm of axon-ensheathing SCs. This provides evidence for the infection of and virus gene expression in neuroglia of the PNS in the clinical phase of RABV encephalitis after i.m. infection.

### Infection of Schwann cells after i.c. inoculation strongly supports anterograde spread of RABV into the periphery

Late phase infection of myelinating SCs in the PNS of i.m.-infected mice could be a result of unspecific virus release from abundantly and long-term RABV-infected myelinated neurons. To investigate, whether RABV also enters the PNS after centrifugal spread from the brain and whether this also leads to infection of myelinating SCs, femoral tissue from i.c.-infected mice was analyzed.

**Fig. 3:**
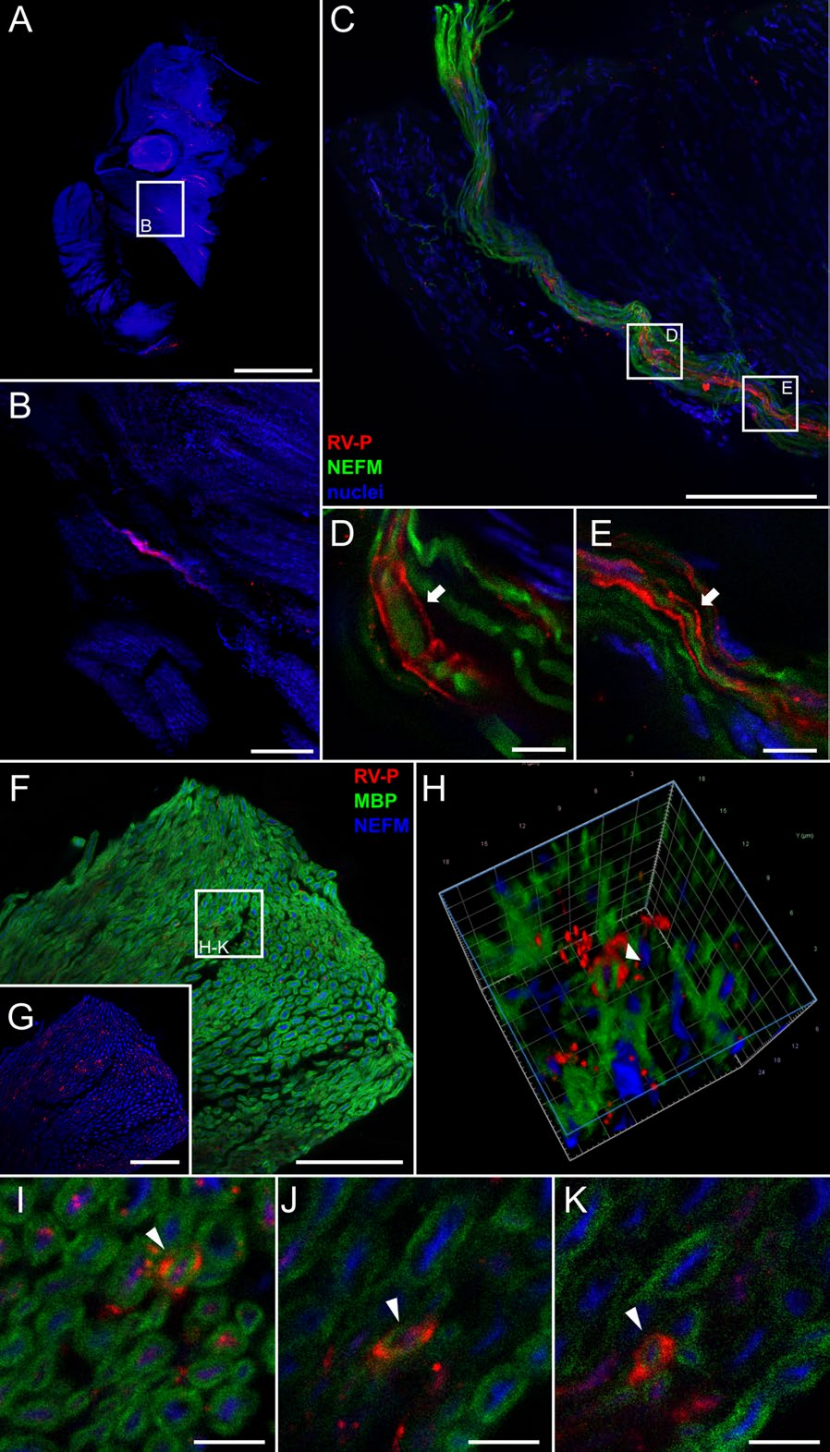
Anterograde virus spread: RABV in Schwann cells of a hind leg nerve after i.c. inoculation. **a**, **b** Maximum z-projection of light sheet overview (**a**) and detail (**b**) of hind leg section after staining for RABV P (red), and nuclei (blue). Green fluorescence for NEFM is not shown as separation from green autofluorescence was not possible at low resolution. **a** Magnification of 1.6x [z = 1944 µm; Scale bar: 1500 µm] and **b** magnification of 12.6x [z = 58 µm; Scale bar: 200 µm]. **c** Maximum z-projection of confocal z-stack [z = 37 µm] of infected nerve fiber from (**b**), now including NEFM staining (green). Scale bar: 100 µm. **d**, **e** Maximum z-projection of details from (**c**) (see white boxes) [z = 5 µm (**d**, **e**)]. Scale bar: 10 µm. **f**, **g** Maximum z-projection of hind leg nerve section stained for RABV P (red), MBP (green) and NEFM (blue) [z = 15 µm]. To visualize low amounts of viral antigen, the MBP channel is excluded in (**g**). Scale bar: 50 µm. **h** 3D projection of detail from (**f**) (see white box). **i–k** Single planes from detail of (**f**). The arrowhead indicates an RABV-infected cell. Scale bar: 5 µm

Detection of RABV antigen in the PNS revealed that innervating nerves in femoral tissue can get infected after centrifugal spread of the virus from the brain (Fig. 3a–g). As observed for i.m. inoculations, RABV P fluorescence signals surrounded rather than overlapped with NEFM-positive axons (Fig. 3d, e). Staining for RABV P, MBP, and NEFM (Fig. 3f–k) revealed a three-layered structure with RABV P-specific signals surrounding the MBP-sheath of axons (Fig. 3h–k, Additional file 5: Video S4). Infection of myelinated SCs after i.c. inoculation demonstrated that infectious virus was released from peripheral axons, infecting the surrounding axon-associated, non-neuronal cells.

### RABV exhibits a strong, infection route-independent infection of the spinal cord

**Fig. 4:**
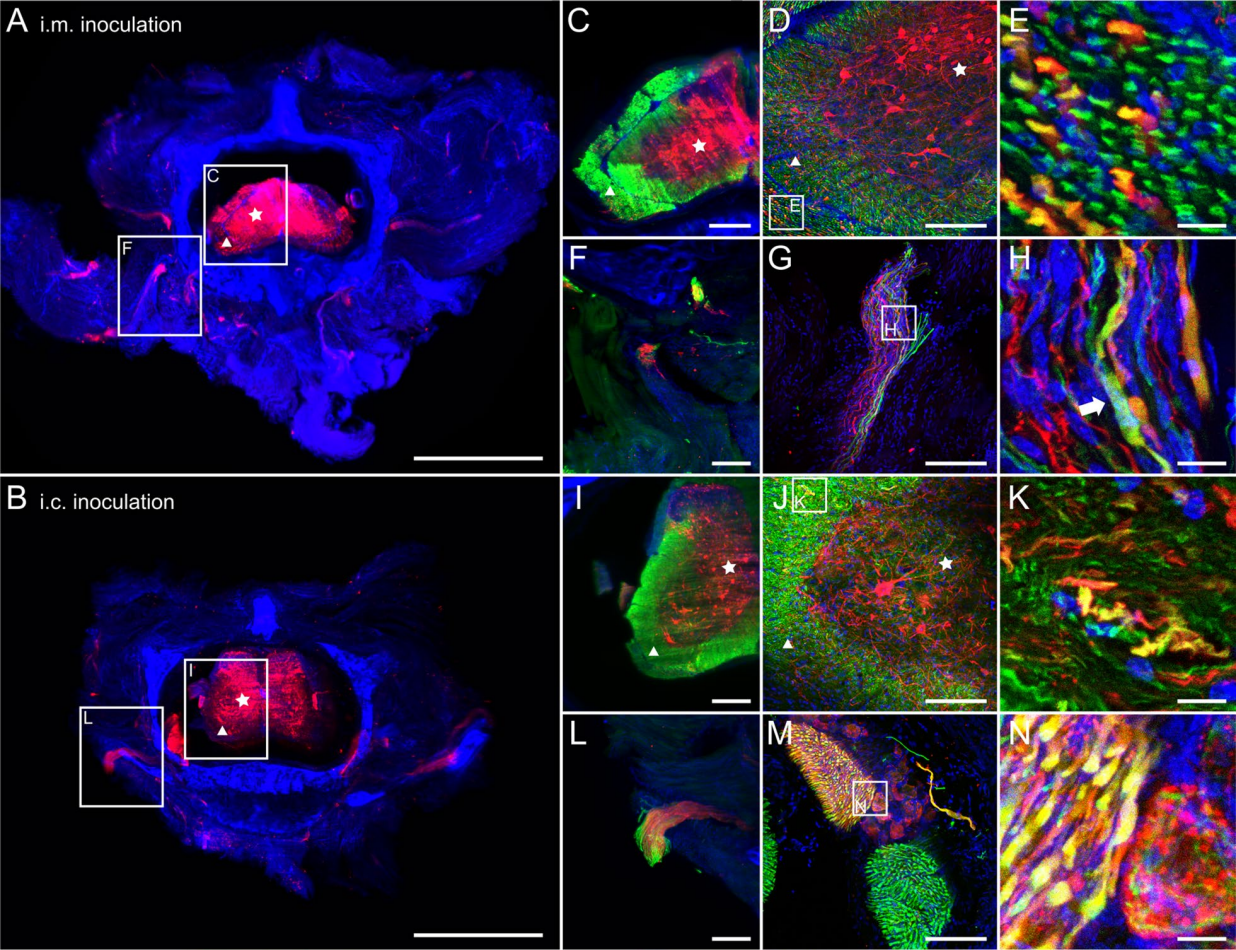
Spinal column infection after i.m. and i.c. infection. **a**, **b** Maximum z-projections of light sheet overviews of cross sections of the spinal column after i.m. (**a**) and i.c. (**b**) infection [2.0x magnification; z = 824 µm (**a**), 1480 µm (**b**)]. Red: RABV P; blue: nuclei. Green fluorescence for NEFM not shown as separation from green autofluorescence was not possible at low resolution. Stars: grey matter, triangles: white matter. Scale bar: 1500 µm. **c** Maximum z-projection of detail from (**a**), including NEFM signals (green). Scale bar: 200 µm. **d**, **e** Maximum z-projection of confocal z-stacks of infected spinal cord (**d**) [z = 31 µm; Scale bar: 100 µm] and detail (**e**) [z = 10 µm; scale bar: 10 µm]. **f** Maximum z-projection of detail from (**a**) with neurons projecting from the spinal cord. Scale bar: 200 µm. **g**, **h** Maximum z-projection of confocal z-stacks (**g**) [z = 36 µm, Scale bar: 100 µm] and detail (**h**) [z = 10 µm; Scale bar: 10 µm] of projecting nerves from the spinal cord. **i** Maximum z-projection of detail from (**b**), including NEFM signals (green). Scale bar: 200 µm. **j**, **k** Maximum z-projection of confocal z-stacks of infected spinal cord (**j**) [z = 61 µm; Scale bar: 100 µm] and detail (**k**) [z = 20 µm; scale bar: 10 µm]. **l** Maximum z-projection of detail from (B), including NEFM signals (green). Scale bar: 200 µm. **m**, **n** Maximum z-projection of confocal z-stacks of projecting nerve (**m**) [z = 41 µm; Scale bar: 100 µm] and detail (**n**) [z = 20 µm; scale bar: 10 µm]

To assess the degree of infection of the spinal cord by field virus clone rRABV Dog after different routes of infection, spinal column slices (Additional file 1: Fig. S2C) of i.m.- and i.c.-infected mice were systematically analyzed. Independent of the inoculation route, multiple neurons in the spinal cord and neurons projecting out of the same were positive for RABV P (Fig. 4).

Higher magnification image stacks of the spinal cord revealed RABV P staining to be primarily localized in the grey matter, surrounded by NEFM-positive white matter (Fig. 4c, i, star and triangle, respectively). Indeed, white and grey matter (Fig. 4d, j, star and triangle, respectively) revealed multiple NEFM-positive axons of which some were infected (Fig. 4e, k). In contrast to the peripheral femoral axons, where the RABV P staining almost exclusively surrounded the tubular NEFM structures (Figs. 2c, d, 3d, e), here, RABV P- and NEFM-specific signals perfectly co-localized (Fig. 4e, k), suggesting that the axons contained high levels of RABV P protein. Peripheral nerves projecting from the spinal cord also revealed double positive axons (Fig. 4f–h, l–n). However, also RABV P not overlapping with NEFM was observed (Fig. 4h, arrow). In some projecting axon bundles (Fig. 4l), almost all axons were RABV-positive as indicated by co-localization of RABV P and NEFM (Fig. 4m, n).

### Pronounced infection of various tissues and sensory neurons of the facial head region

**Fig. 5:**
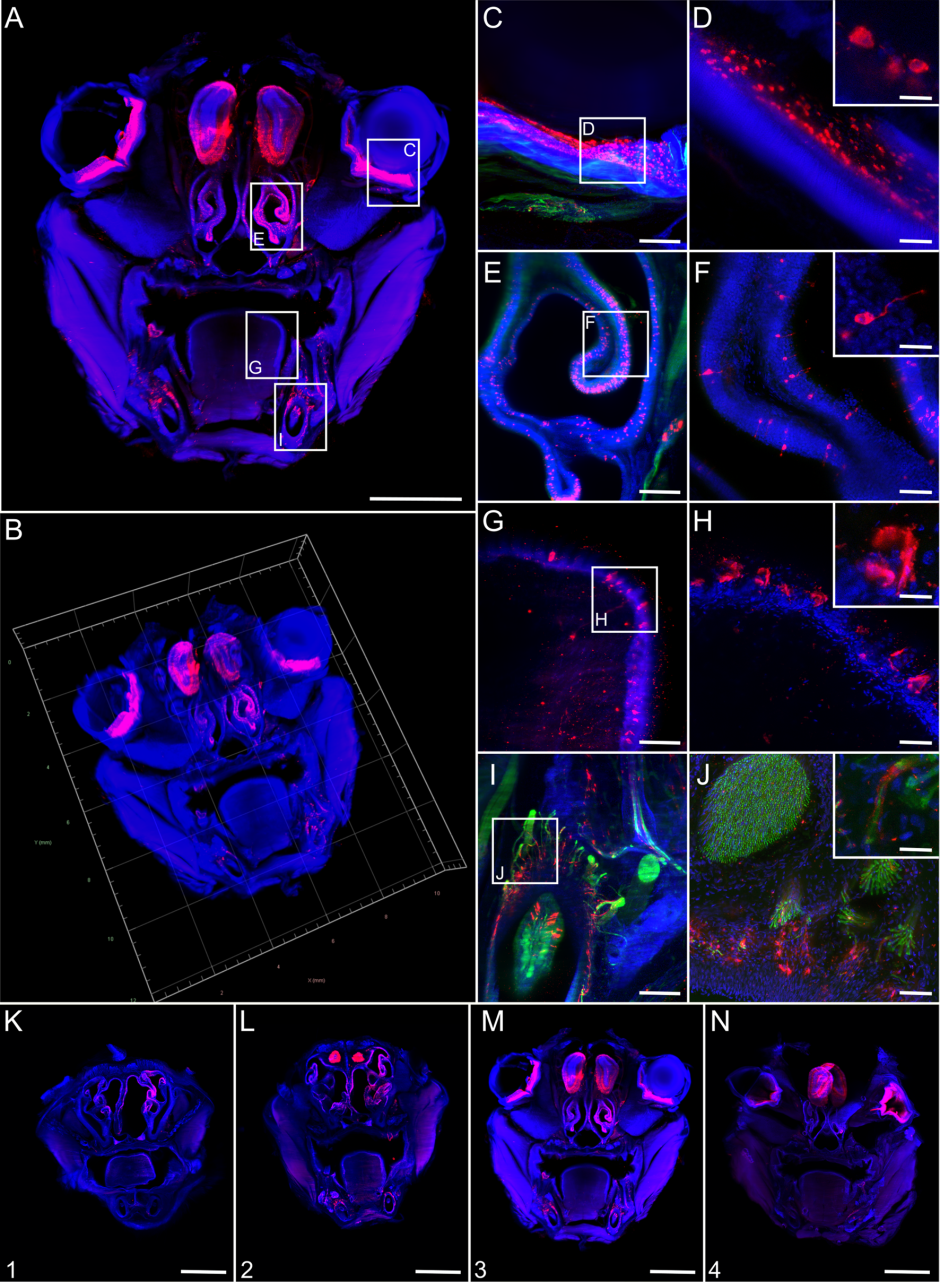
Coronal overview of field RABV-infected mouse head sections after i.c. inoculation. **a**, **b** Maximum z- (**a**) and 3D projection (**b**) of mouse head sections after intracranial infection with field RABV [1.26x magnification; z = 1606 µm]. Indirect immunofluorescence staining against RABV P (red), NEFM (green), and nuclei (blue). Green fluorescence for NEFM not shown as separation from green autofluorescence was not possible at low resolution. Scale bar: 2000 µm. **c**, **e**, **g**, **i** Maximum z-projections of details from A (white boxes) at a magnification of 12.6x. Scale bar: 200 µm. **d**, **f**, **h**, **j** Confocal high-resolution z-stacks (corresponding to the indicated regions in **c**, **e**, **g**, **i**, respectively) [z = 35 µm (**d**), 32 µm (**f**), 36 µm (**h**), 40 µm (**j**); scale bar: 50 µm and 15 µm in detail]. Note: due to the separate confocal image acquisition, the orientations differ from the light sheet microscopy images in **c**, **e**, **g**, and **i**. **c**, **d** Infected eye, **e**, **f** nasal epithelium, **g**, **h** tongue, **i**, **j** and teeth in mouse head coronal section. **k–n** Maximum z-projection of serial mouse head sections (Additional file 1: Fig. S2B) [1.26x; z = 1780 µm (**k**), 1820 µm (**l**), 1606 µm (**m**), 1760 µm (**n**); green not shown]. Scale bar: 2000 µm

**Fig. 6:**
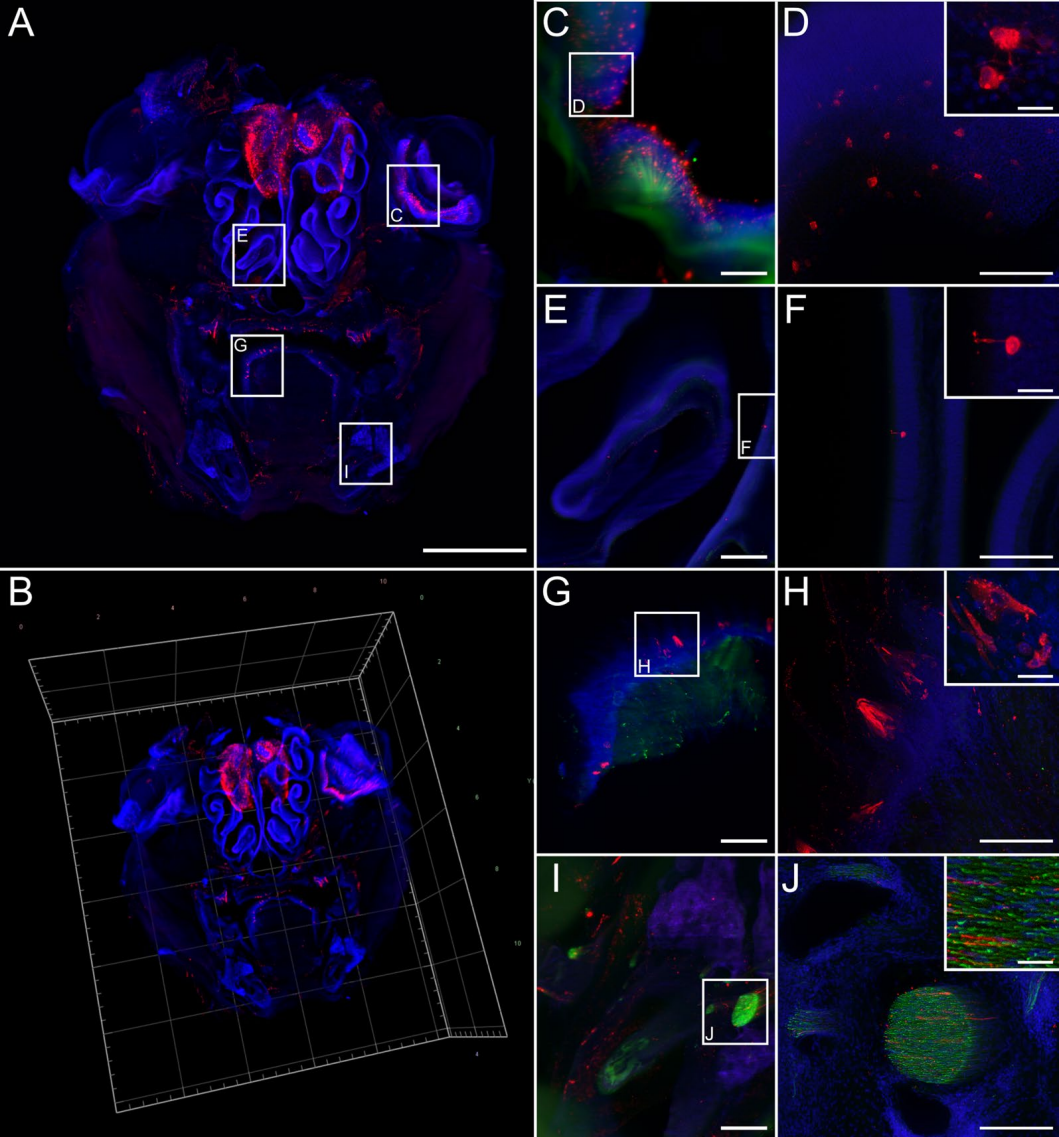
RABV distribution in head cross sections after i.m. inoculation. **a**, **b** Maximum z- (**a**) and 3D projection (**b**) of coronal mouse head sections after i.m. infection with field RABV rRABV Dog [1.26x magnification; z = 4210 µm]. Indirect immunofluorescence staining against RABV P (red), NEFM (green), and nuclei (blue) revealed a markedly reduced degree of infection after i.m. infection in comparison to i.c. infection (see Fig. 5). **c**, **e**, **g**, **i** Maximum z-projections of details from A (white boxes) [12.6x; magnification] (Scale bar: 200 µm). **d**, **f**, **h**, **j** Confocal high-resolution z-stacks (corresponding to **c**, **e**, **g**, **i**, respectively) [z = 80 µm (**d**), 26 µm (**e**), 72 µm (**h**), 74 µm (**j**); scale bar: 100 µm] with details (scale bar: 15 µm). **c**, **d** infected eye, **e**, **f** nasal epithelium, **g**, **h** tongue, **i**, **j** and teeth in mouse head coronal section

Since centrifugal spread to the salivary glands is considered an inevitable step in RABV transmission to other hosts, the extent of RABV infection in tissues of the facial head regions was investigated by imaging of coronal head slices (Additional file 1: Fig. S2B). Except for the olfactory bulb, the brain was removed prior to sectioning for separate processing. 3D volume reconstruction from light sheet microscopy z-stacks demonstrated abundant RABV P-specific signals throughout various areas of the coronal head slice both after i.c. (Fig. 5a, b; Additional file 6: Video S5) and i.m. inoculation (Fig. 6a, b, Additional file 1: Fig. S4; Additional file 7: Video S6). RABV-infected cells could be identified in the orbital (Figs. 5c, d, 6c, d), nasal (Figs. 5e, f, 6e, f), and oral cavity (Figs. 5g–j, 6g–j). Morphologies of the infected cells revealed a structure typical for sensory neurons in the olfactory epithelium and retina (Fig. 5d, f; Additional file 5: Fig. S4), and indicated selective infection of neuronal cells in these tissues. Notably, even though abundant route-independent infection of the olfactory bulb was detectable, presence of fewer RABV-infected cells after i.m. inoculation as compared to the i.c. route demonstrated inoculation route-dependent kinetics of the centrifugal spread to peripheral nerves (Figs. 5a, 6a).

**Fig. 7:**
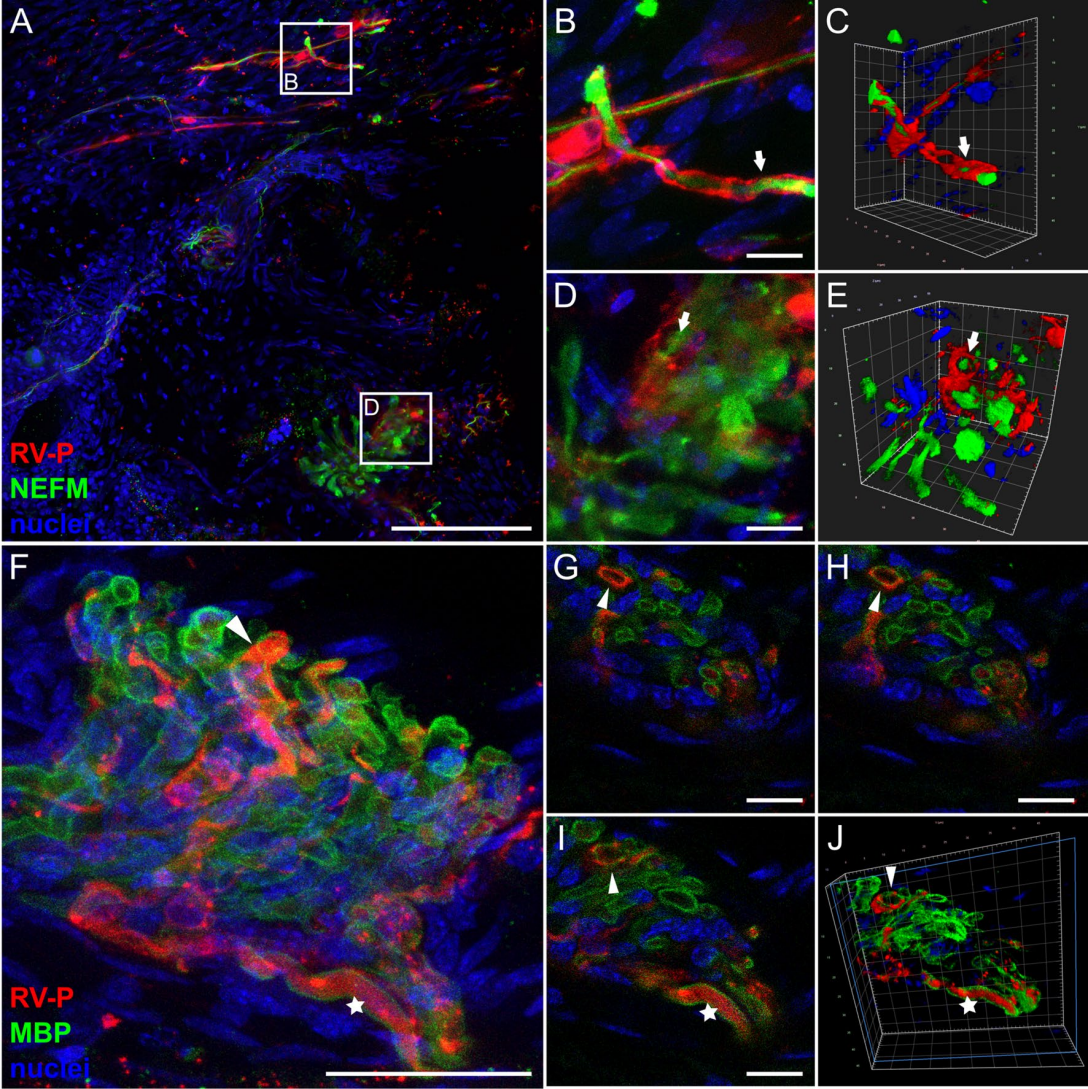
RABV P in axons and infected Schwann cells in coronal head sections. **a** Maximum z-projection of high-resolution confocal z-stacks of mouse head sections after i.c. inoculation and staining for RABV P (red), NEFM (green) and nuclei (blue) [z = 38 µm]. Arrows indicate RABV P surrounding NEFM. Scale bar: 100 µm. **b–e** Maximum z-projections (**b**, **d**) and corresponding 3D projections (**c**, **e**) of details of (**a**) (white boxes). **f** Maximum z-projection of confocal z-stacks of mouse head sections after i.c. inoculation stained for RABV P (red), MBP (green) and nuclei (blue) [z = 29 µm]. Scale bar: 25 µm. **g–i** Single slices of (**f**). Arrowhead: RABV P surrounding MBP-positive myelin sheath. Star: RABV P surrounded by MBP-positive myelin sheath. Scale bar: 5 µm. **j** 3D projection of (**g**–**h**)

Similar to the infection of femoral tissues (Figs. 2, 3), RABV P was arranged around NEFM-positive axons (Figs. 5j, 7a–e), indicating involvement of SCs. Accordingly, RABV was detected at the convex side of the myelin sheaths (Fig. 7g–j, arrowheads; Additional file 8: Video S7). This confirmed infection of neuroglia in RABV infection of peripheral nerves of the facial head region. However, in single cases RABV P was also present inside the myelin sheaths, indicating RABV P accumulation in the respective axons (Fig. 7i, j, stars).

## Discussion

Using organic solvent-based organ clearing [46, 50] for the 3D deep tissue imaging of RABV infections [70], we aimed at investigating and systematically analyzing the in vivo cycle and main checkpoints of a highly virulent field RABV (rRABV Dog) on its journey through the host’s body. To this end, we provide an unprecedented comprehensive overview of the local field RABV tissue tropism and spread in the late clinical phase of RABV encephalitis based on high-volume immunofluorescence imaging. By analyzing peripheral tissues after both i.m. and i.c. infection, we were not only able to visualize RABV infection in PNS and CNS neurons after centripetal and centrifugal spread but also provide clear evidence for the infection of non-neuronal myelinating SCs in peripheral nerves of hind legs and facial head regions (Figs. 2, 3, 7). SCs constitute a glial cell type in the PNS that functionally correlates to myelinating oligodendrocytes in the CNS (reviewed in [20]). Another important finding is the corroboration of anterograde transport and spread of RABV in axons of peripheral nerves by infection of the SCs after centrifugal spread from the CNS.

Myelinating SCs are peripheral neuroglia, which form the myelin sheath around axons of motor and sensory neurons and, like oligodendrocytes in the CNS, are involved in saltatory conduction and trophic support for neurons (reviewed in [52]). In peripheral nerves, RABV infection was associated with axon demyelination [14, 38, 39, 56, 58]. T-lymphocyte-dependent immune pathogenesis was identified to be causative for RABV-mediated neurotic paralysis, the disintegration of myelin sheaths, and the degeneration of axons [66]. Furthermore, there has been speculation that involvement of rabies-induced neuropathogenesis in peripheral nerves results in the typical rabies signs of muscle spasms and deglutition [38].

Although there are indications that SCs are somehow affected by RABV infections [14, 38, 39, 56, 58], evidence for direct RABV infection of SCs in the PNS is lacking. While released RABV particles were detected at nodes of Ranvier (known as myelin-sheath gaps occurring along a myelinated axon [41]) and between axonal and SC plasma membranes [31, 42, 43], only once infection of SCs is described [4]. Others failed to detect RABV infected SCs (reviewed in [51]). Possible explanations for that may be the use of ultrathin sections in electron microscopy in previous studies that run the inherent risk of missing rare events, RABV strain-specific differences in accumulation in peripheral axons and SCs, and variations in the clinical phase of the analyzed animals. Our confirmation that SCs can be infected around peripheral nerves and the ability to visualize it may further support our understanding on the mechanism of Post-exposure prophylaxis (PEP) for rabies. In fact, PEP treatment is highly effective but only when administered in a timely fashion [33]. However, the precise location and mechanisms by which virus neutralizing antibodies interact with viral antigens is not fully understood. Experimental in vivo studies using labelled anti-RABV antibodies followed by our imaging approach may elucidate those processes that lead to the protective effect of pre-formed and elicited antibodies.

SCs are immunocompetent and exert key functions in immune regulation by antigen presentation, pathogen detection, and cytokine production [24, 69]. Accordingly, productive or non-productive infection of these cells could be critically important to immediate innate responses, local inflammatory processes, and the development of adaptive immunity. Recently, we demonstrated that field RABVs, including the recombinant clone rRABV Dog used in this study, are able to infect astroglia in the CNS to a remarkable degree comparable to neuron infections. Whereas infection of astrocytes seems to be a hallmark of field RABVs, more attenuated lab strains are characterized by highly restricted or absent infection of astrocytes. Accordingly, establishment of astroglia infection by field RABVs was hypothesized to limit or block respective immune responses, subsequent virus elimination, and delay immune pathology [49]. RABV infection of SCs in the PNS, as demonstrated in our study (Figs. 2, 3, 7), seems to represent a direct correlate to astroglia infection in the CNS. Comparable to the latter, field RABV replication in peripheral neuroglia may result in local suppression of glia-mediated innate immune responses and, thus, gain the ability to replicate in SCs. In contrast, similar to the astroglia infection in the brain [47, 49], less virulent RABVs may infect SCs in an abortive manner because of a strong type I interferon response.

Evasion of innate and intrinsic antiviral pathways have been attributed to the RABV nucleoprotein (N), P, and matrix (M) proteins [8–10, 37, 63]. As discussed above, there is reason to believe that, similar to current hypotheses about the role of neuroglia infection in the CNS, virus variants are not or only inefficiently able to establish infection in peripheral neuroglia. This may induce stronger local interferon responses, more severe local immune pathogenesis, reduced virus replication, and damage of infected neurons. While we could even confirm infection of SCs by another, bat-associated field RABV (Additional file 1: Fig. S5), analyses with less virulent lab-adapted strains and live-attenuated vaccine viruses have to be subject of further studies to assess whether the virulence-dependent difference in glial cell infection observed in the brain [49] can be transferred to the SC infections in the PNS described in this study.

Virus infection of peripheral SCs in hind legs and facial head regions by centrifugal spread after i.c. inoculation (Figs. 3, 7) as well as infection of sensory neurons in the olfactory epithelium and other tissues (Figs. 5, 6) provide evidence for anterograde transport of RABV along axons in vivo, something that has been attributed to late phases of infection [16]. The long distance from the neuron soma in the respective ganglia and the tight spatial association of the infected neuroglia with the axons makes it exceedingly unlikely that virus release at cell bodies and extra-neuronal spread is responsible for distal SC infections.

Both in vitro and in vivo virus tracking already demonstrated that anterograde RABV transport through axons of peripheral DRG neurons and other peripheral neurons is possible [3, 6, 61]. However, anterograde virus spread was discussed to occur only in a passive, kinesin-independent manner and the release of infectious virus was called into question [62]. Fast, post-replicative, and glycoprotein (G)-dependent anterograde axonal transport of RABV in cultured DRG neurons, however, provided evidence for active transport to distal axon sites [6]. One reason for the discrepancy observed between in vitro tracking and in vivo trans-neuronal tracing approaches could be the role of peripheral neuroglia as major innate immune mediators in infected nerves as discussed earlier. Since the standard neuronal tracer virus CVS-11 is less effective in infecting astrocytes in the brain [49], a strong local antiviral immune response at peripheral sites may hinder CVS-11 replication in neuroglia target cells. Moreover, anterogradely transported virus particles in axons may remain below the limit of detection in conventional in vivo tracing approaches. Accordingly, anterograde transport in CVS-11 infection may have been overlooked because peripheral neuroglia, which were used here as an indicator for anterograde spread of the used field RABV, might not or only abortively get infected.

Beyond RABV spread in peripheral hind leg nerves after i.m. and i.c. infection, a more generalized roadmap of RABV infection in the CNS and PNS could be build. As for human [32, 59] and numerous animal samples (e.g. [28, 29, 35]), abundant RABV-specific signals in the spinal cord and brain could be observed (Fig. 4 and Additional file 1: Fig. S6). Virus detection in the orbital, nasal, and oral cavity confirmed previous reports about presence of RABV and related lyssaviruses in a variety of other tissues [1, 17, 22, 30], including retinal ganglion cells, their axons, and lacrimal glands [15, 25]. Considering the latter, a potential role of lacrimal glands in virus secretion has been discussed [15]. Whereas lacrimal fluid may not contribute to the transmission of infectious virus to new hosts via saliva, multiple infected sensory neurons in the olfactory and tongue epithelia (Figs. 5, 6) with a direct connection to the nasopharynx raise the question whether virus secretion at these sites could contribute to the total amount of infectious virus in the saliva. For bat lyssaviruses, for instance, viral antigen was also detected in taste buds of experimentally [22] and naturally infected animals [53].

## Conclusions

By providing a comprehensive and detailed overview of RABV distribution in peripheral tissues after natural (i.m.) and artificial (i.c.) routes of inoculation, we highlight novel insights in the cell tropism and in vivo spread of field RABV. Importantly, by unveiling the infection of peripheral neuroglia, we suggest a model in which field RABV infection of immunocompetent SCs is critically important for local innate immune responses in peripheral nerves. This may not only contribute to the delayed immune detection of field RABVs through RABV-mediated innate immune response suppression in field virus infected SCs, but may also may explain the high neuroinvasive potential of field RABVs when compared with less virulent lab virus strains, and manifestation of clinical symptoms observed in infected animals and humans. Moreover, by demonstrating infection of myelinating SCs after centrifugal spread from infected brains, we provide evidence for anterograde spread of infectious RABV through axons of the peripheral nerve systems in clinical phases of rabies encephalitis.

## Supplementary information

**Additional file 1: Figures S1 to S6.**

**Additional file 2: Video S1.** Field RABV-infected nerve fibers in mouse hind leg after i.m. inoculation. 3D volume reconstruction of RABV P distribution in femoral tissue (see Fig. 1c in manuscript). 12.6× magnification; z = 574 μm. Red: RABV P; blue: nuclei.

**Additional file 3: Video S2**. Detection of RABV P specific signals around tubular NEFM signals. Tomogram of high-resolution confocal z-stack of RABV-infected nerve fibers. RABV P is not co-localizing with NEFM in axons (arrowheads) but surrounds NEFM positive axons and cells nuclei (arrow). Z = 50 μm. Red: RABV P; green: NEFM; blue: nuclei.

**Additional file 4: Video S3**. RABV P accumulates in the cytomplasm of myelinating Schwann cells. Volumetric 3D projection of infected nerves in hind leg tissue after i.m. inoculation of field RABV. Staining with Schwann cell marker MBP reveals hollow tubules that are surrounded by RABV P. Cell nuclei in the RABV P positive cells further supports of RABV infection of axon ensheating SCs. Red: RABV P; green: MBP; blue: nuclei.

**Additional file 5: Video S4**. Infection of Schwann cells in hind leg nerve after i.c. inoculation. Volumetric 3D projection of RABV P distribution in femoral tissue. Three-layered structure with RABV P-specific signals around axons ensheated by MBP. Red: RABV P; green: MBP; blue: NEFM.

**Additional file 6: Video S5**. RABV infection of various sites in mouse head cross section. 3D volume projection from light sheet microscopy z-stack of field RABV infected mouse head after i.c. inoculation. 1.26× magnification, z = 1606 μm. Red: RABV P; blue: nuclei.

**Additional file 7: Video S6**. Coronal overview of field RABV-infected mouse head section after i.m. inoculation. 3D volume projection from light sheet microscopy z-stack. 1.26× magnification, z = 4210 μm. Red: RABV P; blue: nuclei.

**Additional file 8: Video S7**. Infected Schwann cells in facial head region. Tomogram of high-resolution confocal z-stack of RABV-infected nerve fibers in mouse head after i.c. inoculation. Detection of RABV P at the convex side of the peripheral nerves in mouse head sections (arrowhead). Partially, RABV P is also present inside of myelin sheaths, indicating RABV P accumulation also in the respective axons (arrow). Z = 16 μm. Red: RABV P; green: MBP; blue: nuclei.

## Data Availability

The datasets used and/or analyzed during the current study are available from the corresponding authors on reasonable request.

## Abbreviations

BA: Benzyl alcohol
BB: Benzyl benzoate
CNS: Central nervous system
DCs: Dendritic cells
DPE: Diphenyl ether
DRG: Dorsal root ganglion
EDTA: Ethylenediaminetetraacetic acid
G: Glycoprotein
i.m.: Intramuscular
i.c.: Intracranial
LALFF M-V: State Office for Agriculture, Food Safety, and Fishery in Mecklenburg-Western Pomerania
M: Matrix protein
MBP: Marker myelin basic protein
N: Nucleoprotein
NEFM: Neurofilamtent M
P: Phosphoprotein
PNS: Peripheral nervous system
RABV: Rabies virus
PFA: Paraformaldehyde
RNPs: Ribonucleoproteins
SC: Schwann cell
TBA: *tert*-butanol
